# The microbial peace-signal hypothesis: distributed immune “peace hubs” across the human body

**DOI:** 10.3389/fimmu.2026.1738273

**Published:** 2026-02-06

**Authors:** Cliff Shunsheng Han

**Affiliations:** AllerPops Corp, Los Alamos, NM, United States

**Keywords:** allergies, anaerobes, autoimmunity, hygiene hypothesis, immune tolerance, microbiome, oral microbiota, probiotics

## Abstract

The human immune system depends on microbial partners to maintain restraint. Short-chain fatty acids (SCFAs), produced by anaerobic fermenters in the gut, mouth, and skin, act as biochemical “peace signals” that calm immune activation and promote tolerance. In this hypothesis, “peace signals” refer primarily to microbially derived SCFAs; additional microbial metabolites are discussed as possible but more speculative contributors to immune restraint. This Microbial Peace-Signal Hypothesis proposes that immune homeostasis is not a static legacy of early-life microbial exposure, but a continuous partnership with these commensal fermenters. Modern lifestyle factors—including excessive hygiene, antibiotics, and low-fiber diets—have collapsed the ecological niches that support SCFA-producing guilds. Their loss silences microbial peace signals and drives the epidemic rise of allergies and autoimmune diseases. Unlike the “hygiene” or “old friends” hypotheses, this framework positions microbial peacekeeping as a lifelong metabolic function. It predicts that restoring SCFA producers across all major surfaces—gut, oral, and skin—will reduce immune overactivation systemically. This hypothesis unites clinical, ecological, and evolutionary evidence, suggesting that maintaining distributed SCFA-producing microbiomes is the foundation of long-term immune peace.

## From hygiene to peace signals

In 1989, Strachan proposed the hygiene hypothesis, observing that children from larger families, presumably with more microbial exposure, were less likely to develop hay fever (1). This landmark idea reshaped how researchers viewed the relationship between microbes and immunity.

The concept later evolved into the old friends hypothesis. This model proposed that the rise in allergy and autoimmunity was driven not by reduced infection exposure, but by the loss of evolutionarily ancient microbes—commensal and environmental organisms that had long coexisted with humans. (2).

Later, the immune education model highlighted the importance of microbial interactions in shaping the developing immune system during infancy (3). Gut colonization was seen as essential for “training” T cells, establishing tolerance, and preventing hypersensitivity.

These models were groundbreaking, but all share a central assumption: that the immune system matures into independence after early microbial input. Once educated, tolerance was thought to persist even in the absence of continuous microbial influence.

However, mounting evidence suggests the opposite: the immune system requires ongoing microbial restraint throughout life (4, 5). Without continuous peace signals from microbiota, the immune system defaults to a heightened state of vigilance, prone to misfiring against pollen, food antigens, or self-tissues. This reframing moves beyond hygiene and education, recognizing microbes not as transient tutors but as lifelong partners in immune regulation.

## SCFAs as lifelong peacekeepers

Short-chain fatty acids (SCFAs)—acetate, propionate, and butyrate—are the key microbial signals that restrain immunity. Produced via anaerobic fermentation of dietary fibers, amino acids, and lipids, they: (i) promote regulatory T cell differentiation through HDAC inhibition and epigenetic programming (6); (ii) suppress Th2 and Th17 effector responses that underlie allergy and autoimmunity (6); (iii) inhibit mast cell degranulation (7); and (iv) enhance epithelial barrier function, reducing inappropriate antigen entry (8, 34). Other microbially derived metabolites (such as tryptophan catabolites or bile-acid derivatives) may also modulate immune tone, but current evidence for them as broad, cross-tissue “peace signals” is more limited and remains an area for future investigation (35, 36).

SCFAs are unique because they are metabolized rapidly by colonocytes, hepatocytes, and peripheral tissues (9, 10). They are oxidized for energy, giving them a short half-life. Unlike vitamins or structural microbial components, they cannot be stored. Their signaling value lies in their transience: SCFAs are only present when microbial fermenters are metabolically active.

In other words, immune restraint depends not on past microbial encounters but on continuous microbial peacekeeping in the present moment.

During infection, however, this peacekeeping is deliberately suspended. Microbial activity and SCFA levels decline due to fever, diarrhea during acute infection (11, 12), releasing the immune system from restraint. This allows vigorous responses to eliminate pathogens. Such reversible cancellation underscores the adaptive nature of SCFA signaling: peace in health, but war during infection.

## Dynamic modulation of peace signals

**Figure 1:**
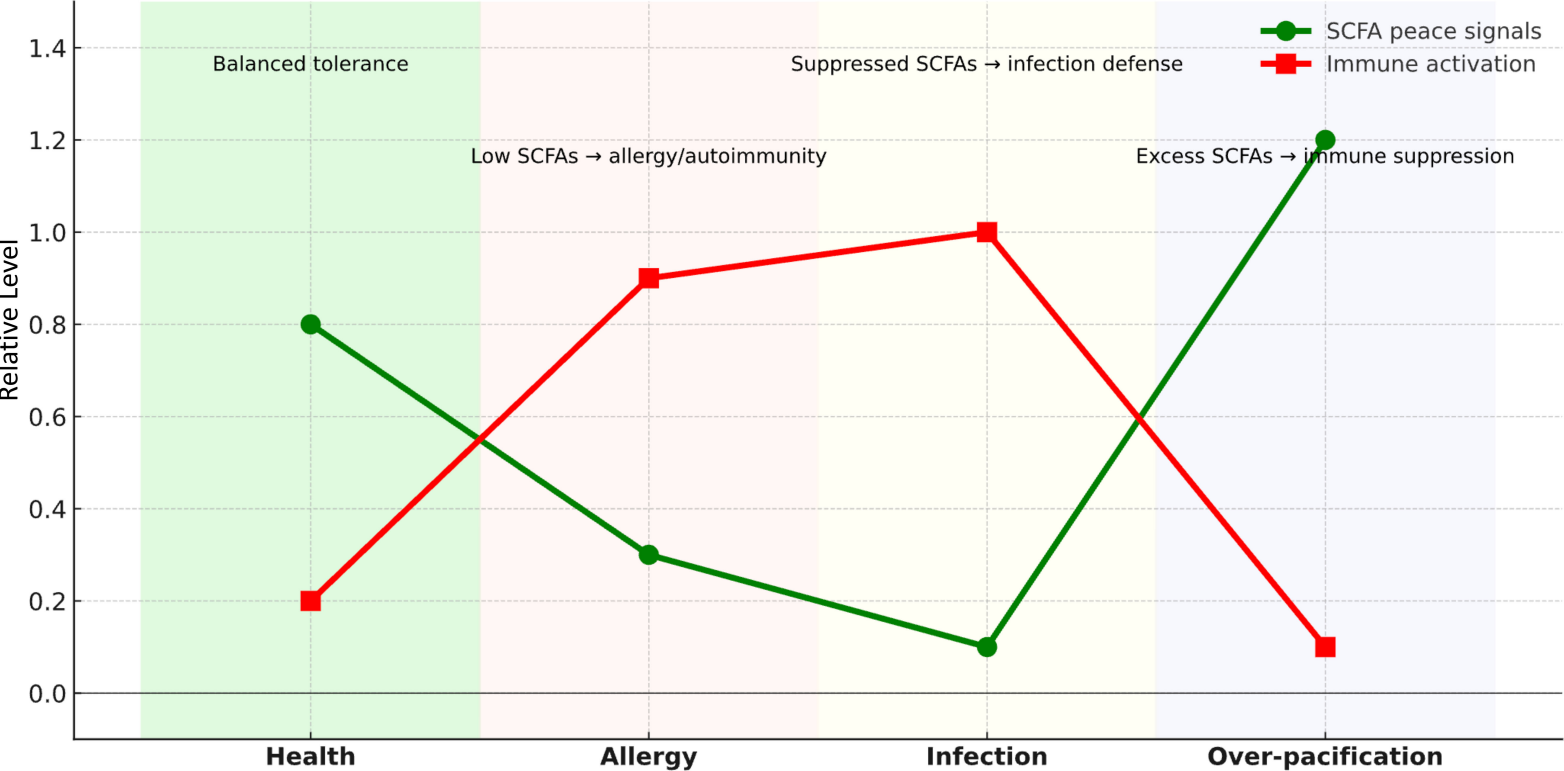
Conceptual model of probiotic–immune modulation across biological conditions. SCFA producers act as real-time peacekeepers: balanced signals sustain tolerance, decline permits hypersensitivity, and deliberate suppression during infection unleashes immunity. Conversely, excessive signaling can over-suppress immunity, increasing infection risk. This dynamic framework highlights the double-edged nature of microbial peacekeeping and explains why the same organisms may appear in both health and infection-associated contexts.

The Microbial Peace-Signal Hypothesis emphasizes that SCFAs are not static background signals but dynamic modulators of immune restraint. Their production and function vary across biological contexts (**Figure 1**).

• Health: Balanced SCFA output maintains tolerance, with regulatory T cells, mast cell suppression, and strengthened epithelial barriers ensuring immune calm.

• Allergy and autoimmunity: Loss or decline of SCFA-producing guilds reduces peace signals, leaving the immune system unrestrained and prone to overreaction against pollen, foods, or self-tissues.

• Infection: SCFA production may be intentionally suppressed during infection (11, 12), canceling peace signals to allow the immune system to fight without restraint. This enables vigorous mechanisms to flush pathogens.

• Over-pacification: Excessive SCFA signaling may over-suppress immunity, increasing vulnerability to pathogens. This may explain why many classical SCFA producers (e.g., *Fusobacterium*, *Peptostreptococcus*) were first identified in infectious pus (13, 14). Here we use “over-pacification” to describe such states of local immune hyporesponsiveness driven by sustained, high SCFA exposure, which can be exploited by pathogens even though the same mechanisms are beneficial in health.

## Distributed peace hubs: gut, mouth, and skin

The gut microbiota remains the most studied SCFA system. Clostridia clusters IV and XIVa induce colonic regulatory T cells (15), Faecalibacterium prausnitzii suppresses pro-inflammatory cytokines (16), and dietary fiber intake correlates with reduced asthma and eczema incidence (17). High-SCFA diets protect against allergic inflammation in animal models, and human cohort studies consistently link low fecal SCFAs to allergy and inflammatory bowel disease (15–17).

**Figure 2:**
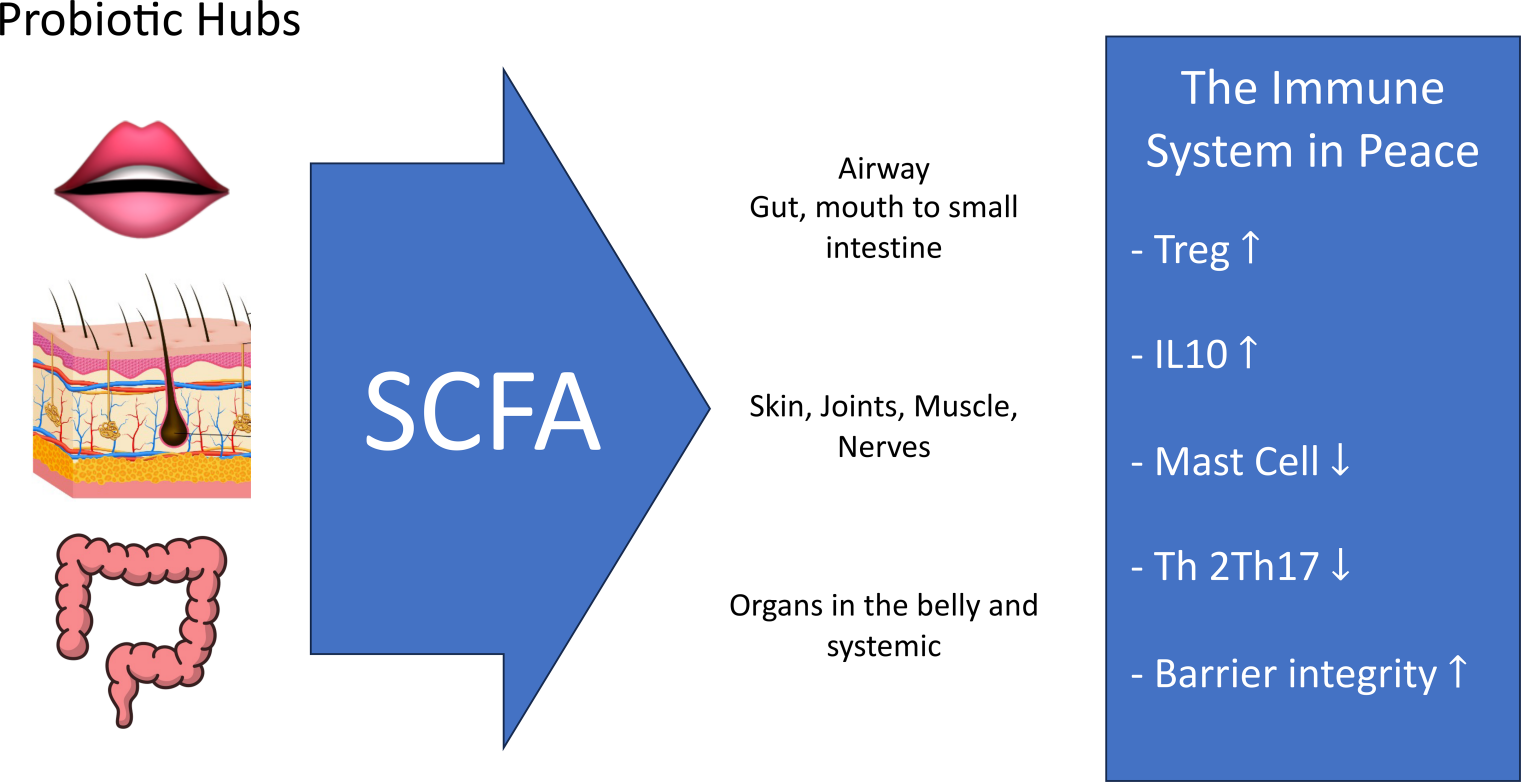
Distributed peace hubs: mouth, skin, and gut → immune restraint. Anaerobic SCFA-producing microbiota in gum pockets and tongue biofilms (mouth), sebaceous follicles (skin), and the colon lumen (gut) generate acetate, propionate, and butyrate. These ‘peace signals’ promote regulatory T cells and IL-10, inhibit mast cells and Th2/Th17 responses, and reinforce barrier integrity—collectively restraining inappropriate inflammation and maintaining immune peace. Cross-tissue effects of oral and skin SCFAs remain speculative and are presented as testable predictions.

Yet the gut is not the only “peace hub,” a term we use for anatomical sites where commensal fermenters generate SCFAs that locally restrain immunity (**Figure 2**). Two other sites—oral and skin microbiota—harbor dense SCFA-producing guilds with potential systemic reach.

### Oral microbiota: gateway to airway peace

The oral cavity harbors anaerobic niches in gum pockets, tongue dorsum biofilms, and tonsillar crypts. Taxa such as *Veillonella*, *Fusobacterium*, *Prevotella*, *Porphyromonas*, and *Peptostreptococcus* thrive in these microenvironments (18–20), and are major contributors to oral SCFA production. Clinical evidence underscores their role: in a randomized controlled trial of allergic rhinitis, a prebiotic oral lozenge restored oral microbial balance. Subjects who improved clinically showed enrichment of *Fusobacteria*, *Butyrivibrio*, and *Peptostreptococcus*—all SCFA producers (21). In a peanut allergy study, salivary butyrate and the abundance of Veillonella nakazawae predicted clinical reactivity thresholds (22). Children with low salivary SCFAs were more sensitive, directly linking oral SCFAs to disease severity.

**Figure 3:**
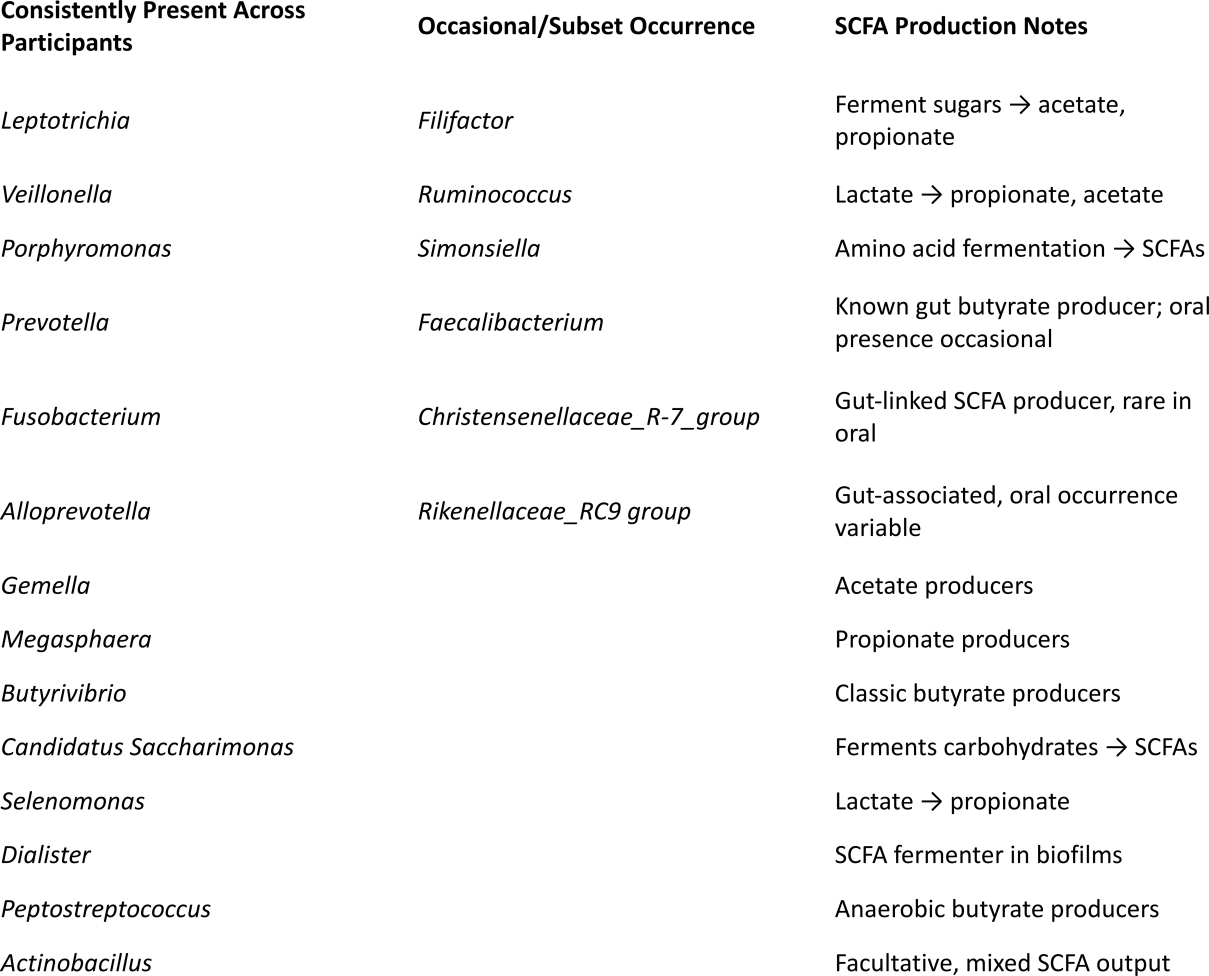
Oral SCFA guild: core and variable producers. The oral cavity contains a functionally redundant guild of SCFA producers. Core taxa observed across all participants include *Leptotrichia*, *Veillonella*, *Porphyromonas*, *Prevotella*, *Fusobacterium*, *Alloprevotella*, *Gemella*, *Megasphaera*, *Butyrivibrio*, *Candidatus Saccharimonas*, *Selenomonas*, *Roseomonas mucosa*, *Dialister*, and *Peptostreptococcus*, with *Actinobacillus* also universally present. Variable taxa (present in subsets) include *Filifactor*, *Ruminococcus*, *Simonsiella*, *Faecalibacterium*, *Christensenellaceae R-7 group*, and *Rikenellaceae RC9 group*. Guild redundancy explains individual variation yet preserves function (SCFA output).

Our cohort findings add detail (**Figure 3**): At least 13 SCFA-producing genera were present across all participants, including *Leptotrichia*, *Veillonella*, *Porphyromonas*, *Prevotella*, *Fusobacterium*, *Alloprevotella*, *Gemella*, *Megasphaera*, *Butyrivibrio*, *Candidatus Saccharimonas*, *Selenomonas*, *Dialister*, and *Peptostreptococcus*, with Actinobacillus universally present. Additional producers such as *Filifactor*, *Ruminococcus*, *Simonsiella*, *Faecalibacterium*, *Christensenellaceae R-7 group*, and *Rikenellaceae RC9 group* appeared in subsets. This cohort consisted of adults with allergic rhinitis; unstimulated saliva samples were profiled by 16S rRNA gene sequencing, and SCFA production was inferred from taxonomic composition rather than directly measured. This diversity suggests that oral tolerance is maintained not by a single keystone species but by a functionally redundant SCFA guild. Just as the gut’s *Clostridia* clusters provide ecological redundancy, the oral cavity relies on overlapping fermenters to maintain immune restraint. Paradoxically, many of these taxa were first identified in infectious pus (13, 14). In certain contexts, their SCFA output may dampen local immunity and allow persistence. This illustrates the double-edged nature of peace signals: indispensable for tolerance, but exploitable by pathogens under specific conditions.

### Skin microbiota: surface peacekeepers

Sebaceous-rich skin regions harbor anaerobic niches where *Cutibacterium* acnes and related taxa ferment sebum lipids into SCFAs (23, 24). These metabolites diffuse into surrounding tissues and calm keratinocytes, dendritic cells, and mast cells (24, 28). Modern hygiene practices—such as frequent washing, antiseptics, and antibiotics—reduce these microbial populations and disrupt SCFA production. Clinical interventions demonstrate the consequences: restoring *Roseomonas mucosa* reduced disease severity in atopic dermatitis and decreased topical steroid use (25, 26). Evolutionary evidence suggests this role is deeply conserved. *Cutibacterium* acnes is abundant not only in humans but also in dogs, cats, and other mammals (27, 28). Its ubiquity across species implies that SCFA-mediated immune regulation at sebaceous sites is an ancient, fundamental strategy in mammalian immune tolerance.

### Cross-hub and systemic influence

SCFAs from each hub likely calm the immune system beyond their immediate niches. Oral SCFAs may benefit the airway mucosa through volatilization and diffusion. For example, acetate is detectable in exhaled breath (32). Swallowed SCFAs may also influence the upper gastrointestinal tract, from the mouth to the small intestine. Skin-derived SCFAs may diffuse into deeper tissues, with evidence that fatty acids and small metabolites can be absorbed transdermally and reach systemic circulation (33). Gut SCFAs, through local diffusion and portal circulation, reach abdominal organs and contribute to systemic immune restraint (34). Similar systemic effects of SCFAs on immune tone have been described in animal and human studies (6). At present, direct mechanistic evidence for cross-tissue effects of oral and skin SCFAs remains limited; these proposed cross-hub influences should therefore be viewed as testable predictions rather than established pathways. This hypothesis provides a framework for understanding their broader physiological reach.

## Collapse of microbial niches in modern life

Why, then, are allergies and autoimmune diseases epidemic in industrialized societies? The Microbial Peace-Signal Hypothesis argues that the root cause is the collapse of microbial niches that sustain SCFA producers.

• Oral hygiene: Aggressive brushing, mouthwash, tongue scraping, and antiseptics disrupt anaerobic guilds in gum pockets and tongue biofilms.

• Skin hygiene: Frequent washing, soaps, and alcohol-based sanitizers strip sebum and commensals.

• Antibiotics: Broad-spectrum treatments indiscriminately eliminate anaerobic fermenters in gut, mouth, and skin.

• Diet: Low-fiber, processed diets deprive gut and oral fermenters of substrates.

• Interruption of early seeding in newborns: Cesarean delivery, reduced breastfeeding, limited skin-to-skin contact, and confinement in highly sanitized hospital environments delay or diminish the transfer of maternal microbiota to infants, weakening the establishment of oral, skin, and gut SCFA guilds during a critical window of immune calibration (29–31).

These pressures collectively erode the ecological homes of SCFA producers. Without continuous peace signals, the immune system reverts to hyper-reactivity, attacking pollen, foods, or self-tissues.

## Predictions and implications

The hypothesis makes specific, testable predictions.

### Biomarkers

- Salivary, skin, and fecal SCFA levels will correlate with allergy and autoimmune disease severity.
- Distinct SCFA guild signatures across individuals will predict resilience or vulnerability.

### Therapeutics

- Restoring oral and skin SCFA producers will improve systemic tolerance, complementing gut-targeted approaches.
- Multi-site prebiotic interventions will outperform gut-only strategies.

### Lifestyle

- Moderate oral and skin hygiene will preserve anaerobic niches.
- Diets rich in fiber and fermented foods will enhance SCFA guild activity.
- Traditional practices such as zuoyuezi (postpartum confinement in Chinese culture), where mother and newborn remain closely together indoors for the first month, may promote early seeding of oral, skin, and gut probiotics. Such intimate contact supports microbial transfer across all three peace hubs, laying the foundation for long-term immune tolerance. Similar evidence supports the benefits of early skin-to-skin contact (29), breastfeeding in microbiota transfer (30), and maternal–infant microbial seeding in shared environments (31).

### Guild redundancy

- Individuals differ in which taxa compose their SCFA guilds, but functional redundancy ensures that tolerance is preserved as long as guild activity is intact.
- Allergy and autoimmunity may arise when redundancy collapses and guild resilience is lost. Here, “guild redundancy” refers to the presence of multiple taxa capable of performing the same SCFA-producing functions, so that loss of individual species does not immediately abolish peace-signal output.

## Paradigm shift

The hygiene hypothesis reframed infection avoidance as a risk factor. The old friend hypothesis emphasized co-evolutionary microbial partners. The immune education model highlighted infancy as a critical window.

The Microbial Peace-Signal Hypothesis extends these by asserting that: (i) immune restraint is not a one-time event but a lifelong microbial function; (ii) SCFAs serve as dynamic peace signals, continuously consumed, continuously renewed; (iii) guild redundancy across gut, mouth, and skin is essential, ensuring resilience but also explaining differential susceptibility; and (iv) infection control requires temporary suspension of peace signals, while microbial misuse can flip tolerance into persistence. This reframing unifies allergy and autoimmunity under a single principle: both are consequences of collapsed microbial peacekeeping systems.

## Conclusion

Immune homeostasis is not a static legacy of early-life microbial encounters but a continuous partnership with commensal fermenters. SCFAs act as biochemical peace signals, transient yet powerful, restraining immunity in real time. When the guild of SCFA producers thrives—in gut, mouth, and skin—the immune system remains calm. When modern life erodes these guilds, peace signals fall silent, and immunity overreacts. The Microbial Peace-Signal Hypothesis reframes allergy and autoimmunity as failures of microbial–immune symbiosis. It highlights diagnostics, lifestyle practices, and therapeutic strategies that restore microbial peace as the next frontier in immunology.

## Data Availability

The original contributions presented in the study are included in the article/supplementary material. Further inquiries can be directed to the corresponding author.
